# The relationship between pre-operative lymphocyte to monocyte ratio and serum cancer antigen-125 among women with epithelial ovarian cancer in Lagos, Nigeria

**DOI:** 10.3332/ecancer.2021.1288

**Published:** 2021-09-14

**Authors:** Adaiah Priscilla Soibi-Harry, Lemchukwu Chukwunonye Amaeshi, Sunusi Rimi Garba, Rose Ihuoma Anorlu

**Affiliations:** 1 Oncology & Pathological Studies Unit, Department of Obstetrics & Gynaecology, Lagos University Teaching Hospital, Lagos 101233, Nigeria; 2 Clinical Haematology and Oncology Unit, Department of Medicine, Lagos University Teaching Hospital, Lagos 101233, Nigeria; 3 Department of Obstetrics & Gynaecology, University of Lagos/Lagos University Teaching Hospital, Lagos 101233, Nigeria

**Keywords:** ovarian cancer, lymphocytes, monocytes, CA-125

## Abstract

Ovarian cancer (OC) is the second most common genital cancer worldwide, and the most lethal of all genital cancers. The role of inflammation and markers of systemic inflammation such as neutrophils, lymphocytes and monocytes in cancer biology have been investigated and reported in many studies. Cancer antigen 125 (CA-125) is currently in use as an adjunct to diagnosis, prognostication and monitoring of epithelial OC (EOC). This test is not readily available in many centres in sub-Saharan Africa, creating a need to identify alternative markers that are available and affordable. This study aimed to determine the relationship between pre-operative serum lymphocyte to monocyte ratio (LMR) and CA-125 in EOC. This was a retrospective cross-sectional study among 70 women, diagnosed with EOC in Lagos University Teaching Hospital from January 2013 to December 2019. Data were extracted from the case notes of the patients. LMR was calculated as the absolute lymphocyte count divided by the absolute monocyte count and analysed using Statistical Package for Social Sciences (SPSS) version 25.0. The correlation between LMR and CA-125 was determined using Pearson’s correlation coefficient. The mean age of the patients was 48.57 ± 13.97 years. Serous adenocarcinoma was the most common subtype of EOC making up 94.3% of the cases. The median serum CA-125 was 393.5 (215.00–765.67) U/mL. The median LMR was 6.77 (1.28–43.0). There was a statistically significant negative correlation between CA-125 and LMR, *r* = −0.28, *p* = 0.02. LMR was negatively associated with CA-125 in women with EOC. LMR may be considered as a simple, affordable alternative marker to CA-125 in the management of EOC.

## Background

Ovarian cancer (OC) is second only to cervical cancer as the most common cause of gynaecologic cancer deaths globally [1]. It is the most lethal of all gynaecological cancers. In most developed countries, it is the leading cause of gynaecologic cancer deaths with a life time risk of 1 in 70 [2, 3]. In Nigeria, several studies have shown a general trend towards an increase in the incidence of OC countrywide. Studies on OC from different centres in Nigeria found it constitutes 7%-26% of all gynaecological malignancies [4]. It was the second most common cause of death on the gynaecology wards in Lagos University Teaching Hospital, Lagos, Nigeria [5]. In Nigeria, many of the centres that treat OC are found in urban areas.

Cancer antigen 125 (CA-125) is the most widely used marker in the management of patients with epithelial OC (EOC), and can be used as a predictive factor of overall survival (OS) in patients with EOC [6]. Since the time of Rudolf Virchow, who studied solid tumours and their microenvironment, several studies have shown that inflammation is associated with cancer and it is considered the seventh hallmark of cancer [7]. Inflammatory markers such as neutrophil to lymphocyte ratio (NLR), platelet to lymphocyte ratio (PLR) and lymphocyte to monocyte ratio (LMR), derived from haematological indices, have been studied in several malignancies such as breast cancer, gastro-intestinal malignancies and OC. They have not only been found to be elevated in these malignancies, but have also been found to be independent predictive and prognostic factors in determining OS [8–10].

The search for prognostic and predictive tools for OC has continued, with recent studies focusing on the relationship between OC and systemic inflammatory response. Some studies have shown that NLR, monocyte to lymphocyte ratio and PLR may be useful to predict clinical outcomes in patients with EOC [8, 11–13]. CA-125 has been found to be an independent predictive and prognostic factor in determining OS in EOC [14].

Markers of systemic inflammation such as neutrophils, lymphocytes, monocytes can be easily measured, are relatively cheap, readily available even in rural settings in Nigeria, affordable, reproducible and cost-effective. CA-125 is generally used in the monitoring of patients with EOC in Nigeria. It is unaffordable for many patients in Nigeria as they pay out of pocket to access healthcare and this usually leads to disruption of treatment and loss to follow-up. We, therefore, set out to determine the relationship between pre-operative LMR, an inflammatory biomarker, and CA-125 in women with EOC.

## Materials and methods

This was a retrospective cross-sectional study. The medical records of women who underwent staging laparotomy for suspected OC at the Lagos University Teaching Hospital, Nigeria during the period 1 January 2013–31 December 2019 were retrieved from the Medical Records Unit. A structured form was used to extract information on age, parity, menopausal status, histologic sub-type, The International Federation of Gynaecology and Obstetrics (FIGO) stage, complete blood count result and CA-125 levels. Patients with other co-existing cancers, autoimmune disease, evidence of sepsis and those who had neo-adjuvant chemotherapy were excluded from the study. Histologic type classification was reviewed by a single pathologist.

The LMR was calculated as the absolute count of lymphocytes divided by the absolute count of monocytes. Data were analysed using SPSS version 25.0, IBM Corp., Armonk, NY, USA. The correlation between LMR and CA-125 levels was determined using Pearson’s correlation coefficient.

Ethical approval was obtained from the Health Research and Ethics Committee of the Lagos University Teaching Hospital.

## Results

A total of 101 laparotomies for ovarian tumours were performed during the period. Data were available for analysis in 98 patients, of which 70 had EOC.

### Baseline characteristics of patients

Table 1. shows the baseline characteristics of the 70 patients. The mean age was 48.57 ± 13.97 years, and about three-quarters of the patients were above the age of 40 years. Over 50% of patients were post-menopausal and serous adenocarcinoma was the most common histological sub-type 66/70 (94.3%).

### Haematological parameters of patients with EOC

The median CA-125 level and the haematological parameters of patients with EOC are shown in Table 2. The median CA-125 was 393.50 U/mL.

### Relationship between CA-125 and LMR

**Figure 1.**
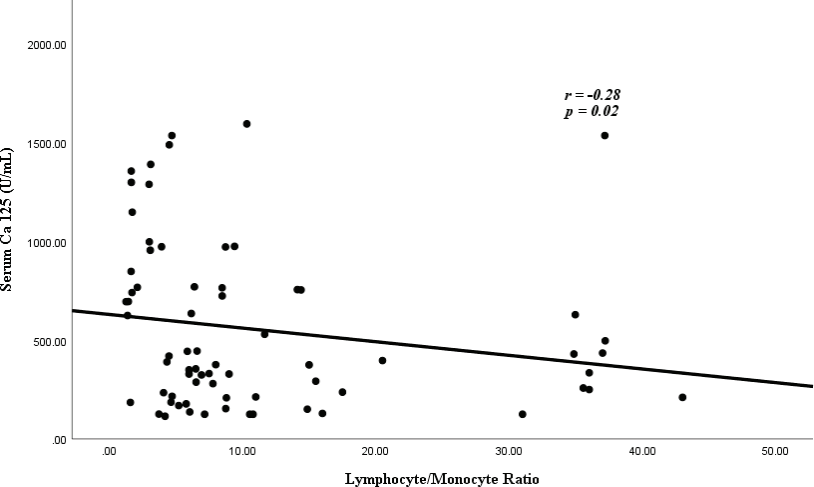
Correlation of serum CA-125 and LMR.

Figure 1 shows the correlation between CA-125 and LMR. There was a statistically significant, negative correlation between serum CA-125 levels and LMR (*r* = −0.28, *p* = 0.02).

## Discussion

Our study found a statistically significant negative correlation between CA-125 and LMR in women with EOC. This implies that low LMR was associated with high serum CA-125 levels in women with EOC. CA-125, also known as Muc16, is a mucin-type O-linked glycoprotein of high molecular mass whose exact role in health and disease process is not well understood. However, it is currently in use as an adjunct to diagnosis, in the monitoring of response to therapy, detection of recurrence of disease and determination of prognosis in EOC. Although a nonspecific marker of disease, it has been found to be elevated in 50% of women with early stage EOC and 80% of women with advanced disease [14–17].

A low LMR indicates a decrease in lymphocyte and/or increase in total monocyte count.

Lymphocytes are known to play a role in cellular and humoral anti-tumour immune responses [18].

Activated and proliferating lymphocytes play a role in cytotoxic cell death and inhibit tumour cell proliferation and migration. Many studies have demonstrated the positive role lymphocytes play in the response of tumours to chemotherapy [19–21].

Clinical and experimental studies indicate that macrophages may promote solid-tumour progression and metastasis [22, 23]. These tumours associated macrophages are considered to arise primarily from monocytes. They seem to promote proliferation, invasion and metastasis of tumour cells, stimulate tumour angiogenesis and inhibit antitumor immune response mediated by T cells, followed by the promotion of tumour progression. A high monocyte count is therefore indirectly associated with tumour progression [24].

Our study found a statistically significantly negative correlation between CA-125, a tumour marker that is elevated in EOC, and LMR. A high serum monocyte level as reflected in low LMR was associated with high CA-125 levels. In two separate meta-analysis involving women with OC, a low LMR predicted shorter OS [25, 26].

The statistically significant negative correlation between LMR and CA-125 level, that we found in our study, indicates that LMR might be a potential surrogate for CA-125 in monitoring of patients with EOC during treatment and follow-up of their disease. In Nigeria, full blood count is available even in rural settings and cost about 2,000 naira (US$ 4.25), whereas CA-125 is only available in special laboratories in the urban areas and costs 8,000 naira (US$ 17.00). Therefore, this relationship between LMR and CA-125 levels can be explored further as an affordable and readily available tool in monitoring treatment of patients with EOC in the same way as CA-125. Some of the limitations of this study include the relatively small number of cases, the fact that white blood cell count levels may be affected by the presence of other disease conditions and that post operatively, CA-125, lymphocyte and monocyte levels may change.

## Conclusion

In conclusion, pre-operative low LMR is associated with high CA-125 in women with EOC and perhaps could be considered as a potential simple, available, affordable, convenient and reproducible alternative tool in monitoring of women with EOC in resource poor countries.

## Conflict of interest

There is no conflict of interest.

## Funding

We did not receive any funding for this study.

## Figures and Tables

**Table 1.** Baseline characteristics of patients with EOC (*N* = 70).

Variables	*n*	%
Age (years)		
<30	6	8.6
30–39	14	20
40–49	14	20
50–59	15	21.4
>60	21	30
Mean age (years) ± SD	48.57 ± 13.97	
Parity		
0	20	28.6
1–2	11	15.7
3–4	21	30
≥5	18	25.7
Mean parity (Mean ± SD)	2.87 ± 2.40	
Menopausal status		
Premenopausal	33	47.1
Post-menopausal	37	52.9
Histological sub-type		
Serous	66	94.3
Mucinous	4	5.7
FIGO staging		
Stage 3	21	30
Stage 4	49	70

**Table 2.** Haematological indices of patients with serous histotype of EOC (N = 66).

Variables	Median	IQR
Haematocrit (%)	33.00	29.63–36.10
Platelet count (10^9^/L)	303.50	217.75–417.50
White blood cell count (10^9^/L)	6.65	5.00–8.56
Lymphocyte count (10^9^/L)	2.05	1.41–2.67
Monocyte count (10^9^/L)	0.29	0.16–0.49
LMR	6.77	4.17–14.19
CA-125 (U/mL)	393.50	215.00–765.67

IQR, Interquartile range
